# *Streptococcus pneumoniae* Affects Endothelial Cell Migration in Microfluidic Circulation

**DOI:** 10.3389/fmicb.2022.852036

**Published:** 2022-03-25

**Authors:** Anna Kopenhagen, Isabell Ramming, Belinda Camp, Sven Hammerschmidt, Marcus Fulde, Mathias Müsken, Michael Steinert, Simone Bergmann

**Affiliations:** ^1^Institut für Mikrobiologie, Technische Universität Braunschweig, Braunschweig, Germany; ^2^Department of Infectious Diseases, Robert Koch Institute, Wernigerode, Germany; ^3^Department of Pneumology, University Hospital Magdeburg, Magdeburg, Germany; ^4^Institute for Genetics and Functional Genomics, Department of Molecular Genetics and Infection Biology, Universität Greifswald, Greifswald, Germany; ^5^Institute of Microbiology and Epizootics, Department of Veterinary Medicine, Freie Universität Berlin, Berlin, Germany; ^6^Central Facility for Microscopy, Helmholtz Centre for Infection Research, Braunschweig, Germany; ^7^Helmholtz Centre for Infection Research, Braunschweig, Germany

**Keywords:** *Streptococcus pneumoniae*, endothelium, cell migration, microfluidic, wound healing, pneumolysin

## Abstract

Bloodstream infections caused by *Streptococcus pneumoniae* induce strong inflammatory and procoagulant cellular responses and affect the endothelial barrier of the vascular system. Bacterial virulence determinants, such as the cytotoxic pore-forming pneumolysin, increase the endothelial barrier permeability by inducing cell apoptosis and cell damage. As life-threatening consequences, disseminated intravascular coagulation followed by consumption coagulopathy and low blood pressure is described. With the aim to decipher the role of pneumolysin in endothelial damage and leakage of the vascular barrier in more detail, we established a chamber-separation cell migration assay (CSMA) used to illustrate endothelial wound healing upon bacterial infections. We used chambered inlets for cell cultivation, which, after removal, provide a cell-free area of 500 μm in diameter as a defined gap in primary endothelial cell layers. During the process of wound healing, the size of the cell-free area is decreasing due to cell migration and proliferation, which we quantitatively determined by microscopic live cell monitoring. In addition, differential immunofluorescence staining combined with confocal microscopy was used to morphologically characterize the effect of bacterial attachment on cell migration and the velocity of gap closure. In all assays, the presence of wild-type pneumococci significantly inhibited endothelial gap closure. Remarkably, even in the presence of pneumolysin-deficient pneumococci, cell migration was significantly retarded. Moreover, the inhibitory effect of pneumococci on the proportion of cell proliferation versus cell migration within the process of endothelial gap closure was assessed by implementation of a fluorescence-conjugated nucleoside analogon. We further combined the endothelial CSMA with a microfluidic pump system, which for the first time enabled the microscopic visualization and monitoring of endothelial gap closure in the presence of circulating bacteria at defined vascular shear stress values for up to 48 h. In accordance with our CSMA results under static conditions, the gap remained cell free in the presence of circulating pneumococci in flow. Hence, our combined endothelial cultivation technique represents a complex *in vitro* system, which mimics the vascular physiology as close as possible by providing essential parameters of the blood flow to gain new insights into the effect of pneumococcal infection on endothelial barrier integrity in flow.

## Introduction

*Streptococcus pneumoniae* is an opportunistic pathogen colonizing the nasopharyngeal epithelium of the upper respiratory tract of humans and other mammals. *S. pneumoniae* (the pneumococcus) is also listed as one of the most frequent microbial causes of fatal infections in non-pandemic times by the World Health Organization (Abdullahi et al., 2008; World Health Organization, 2017). In children and immunocompromised and elderly individuals, pneumococci cause a broad spectrum of local infections such as otitis media, sinusitis, and severe invasive diseases including lobar pneumonia, meningitis, and septicemia (Bogaert et al., 2004; Kadioglu et al., 2008; Henriques-Normark and Tuomanen, 2013; Valles et al., 2016). Despite the implementation of a 13-valent pneumococcal conjugate vaccine (PCV), the morbidity and mortality rates remain at rather high levels, especially in the elderly and the immunocompromised subpopulation (Kadioglu et al., 2008; Imohl et al., 2015; Valles et al., 2016).

During systemic infection, pneumococci attach *via* surface-displayed adherence factors to endothelial cells and utilize endothelial cell surface receptors for colonization and transmigration (Bergmann et al., 2001, 2003; Tasaka et al., 2003; Bergmann and Hammerschmidt, 2006; Iovino et al., 2014a,b, 2016). Moreover, pneumococci produce the cholesterol-dependent pore-forming cytotoxin pneumolysin, which is a key virulence factor inducing cell death by pore formation and toxin-induced apoptosis (Marriott et al., 2008). Pneumolysin affects different cell types, e.g., endothelial cells of the vascular system, tracheobronchial epithelial cells, and platelets, and contributes massively to tissue damage in acute lung infections by also interfering with the eukaryotic signal transduction and by modulating the epigenetic modification (Witzenrath et al., 2006; Marriott et al., 2008; Mitchell and Dalziel, 2014; Jahn et al., 2020).

In addition, pneumococci subvert the host-derived plasmin activity to cleave intercellular junction proteins, which facilitates bacterial crossing of both epithelial and endothelial barriers (Bergmann et al., 2005, 2013; Bergmann and Hammerschmidt, 2007; Attali et al., 2008; Peter et al., 2017). The acute inflammatory host responses, which are induced by the concerted action of several pneumococcal pathomechanisms and virulence factors, promote collateral endothelial cell damage and consequently lead to an increased vascular permeability (Van der Flier et al., 2001; Loughran et al., 2019). Endothelial cells constitute the luminal surface of all blood and lymph vessels. The major function of endothelial cells includes the control of vascular permeability and the mediation of vascular response toward inflammation induced by injury or infection (Pober and Sessa, 2007; Reinhart-King, 2008). The loss of endothelial barrier integrity during systemic bacterial infections in turn triggers the induction of vascular regeneration *via* cell migration and cell proliferation (Broughton et al., 2006; Reinhart-King, 2008; Michaelis, 2014; Joffre et al., 2020). In contrast to the already well-described mechanisms of endothelial cell migration in general, the pathophysiological effects of pneumococcal interaction with the endothelial cells with respect to migration and proliferation are not yet clarified in detail. Most of the experimental cell migration analyses, which are commonly referred to as “wound healing assays,” reported so far were focused on epithelial tissues foremost with respect to cutaneous wound healing, embryogenesis, and tumor metastasis (Singer and Clark, 1999; Ilina and Friedl, 2009; Vedula et al., 2013; Jonkman et al., 2014). In the majority of performed assay techniques, a proportion of cells are initially removed by mechanical, thermal, or chemical damage of a confluently grown cell monolayer to create a cell-free area as starting point for cell migration (Ilina and Friedl, 2009; Vedula et al., 2013; Jonkman et al., 2014).

Shear stress, which is generated by the hemodynamic forces of the bloodstream on vascular cells, is known to significantly modulate cellular proliferation and migration (Rousseau et al., 2000; Chistiakov et al., 2017). Only recently, it became evident that shear forces are sensed by special protein complexes named endothelial mechanosomes, which induce a shear stress-dependent modification of intracellular signal transduction *via* structurally dynamic protein domains (Chistiakov et al., 2017). This leads, for example, to an increase in the expression of surface proteins that are necessary for the formation of strong cell–cell contacts and for morphological characteristics in general as well as for functional barrier properties of the cell layer (Tzima et al., 2005; Ting et al., 2012; Chistiakov et al., 2017). In former studies, we established an infection cell culture model using a microfluidic pump system for the characterization of pneumococcal attachment to endothelial cells in flow (Jagau et al., 2019a,b). This microfluidic system enables the simulation of different shear stress scenarios typically present under physiological and pathological conditions in the human blood stream.

The process of endothelial tissue recovery under shear stress during bacterial infection and the impact of pneumococcal virulence factors on the velocity and efficacy of endothelial wound healing under higher shear forces are not clarified in detail yet. Here, we describe a chamber-separation cell migration assay (CSMA) based on a silicone inlet system of ibidi^®^, which enables a microscopic real-time monitoring of endothelial cell migration and also a precise quantification of the velocity of cell migration, as well as of bacterial cell attachment, and a characterization of cell morphology. Furthermore, we combined the CSMA with our formerly established microfluidic pump system to mimic the flow parameter present in bloodstream infections and to analyze the impact of a defined shear force on endothelial cell migration during pneumococcus infection.

## Materials and Methods

### Cultivation of Bacteria and Human Endothelial Cells

Infections were performed with *Streptococcus pneumoniae* serotype 35A (ATCC11733, Jagau et al., 2019a), since this serotype is less capsulated and is described as a suitable serotype producing the whole subset of virulence traits (Bergmann et al., 2009; Luttge et al., 2012). This strain henceforth is abbreviated as WT. In addition, we use the isogenic pneumolysin-deficient isogenic strain (St35AΔ*ply*, Pracht et al., 2005). Pneumococci were grown to mid log phase in Todd Hewitt liquid broth supplemented with 5% yeast extract at 37°C and 5% CO_2_. The Δ*ply* strain was cultivated in the same medium supplemented with 5.0 μg/ml erythromycin. To infect endothelial cells, bacteria were centrifuged for 4 min at 3,000 × *g*, washed twice with 10 ml phosphate-buffered saline (PBS), and an amount of 1 × 10^8^ pneumococci per mL was photometrically adjusted by determining the optical density at 600 nm. For heat inactivation of pneumococci, 1 × 10^9^ bacteria were resuspended in 1 ml PBS and incubated at 60°C for 1 h. Heat inactivation was confirmed by plating on blood agar.

Human umbilical vein endothelial cells (HUVEC) were purchased as pooled donor cryostocks from PromoCell (Heidelberg, Germany) and were subcultivated using Accutase solution according to the recommendations of the manufacturer. The primary endothelial cells were cultivated in Endothelial Cell Growth Medium (ECGM, PromoCell) supplemented with the recommended suspension of growth factors and hormones in 75-cm^2^ cell culture flasks at 37°C and 5% CO_2_. Subcultured cells were used only up to the 8th passage.

### Proteins and Antibodies

Pneumococcus-specific polyclonal antibodies were generated in rabbit by Pineda. Alexa Fluor 488-conjugated goat anti-rabbit, Alexa Fluor 568-conjugated goat anti-rabbit, and 4′,6-diamidino-2-phenylindole (DAPI) were from Thermo Fisher Scientific (Waltham, MA, United States), paraformaldehyde (PFA) was purchased from Polysciences (Hirschberg, Germany), and mounting medium was from Dako (Jena, Germany). Alexa-488-conjugated phalloidin was purchased from Abcam (Cambridge, MA, United States) and porcine gelatin from Merck (Burlington, MA, United States). Purification of Strep-tagged pneumolysin was described recently (Jahn et al., 2020). Pneumolysin-activity analyses were performed using citrated sheep blood from Fiebig Nährstofftechnik, Germany. In addition, the purity and integrity of the purified pneumolysin protein were also controlled by denaturating SDS-polyacrylamide gel electrophoresis.

### Hemolysis Analyses

The hemolytic activity of pneumococcal lysates was determined essentially as described by Benton et al. (1997). In brief, heterologously produced and purified pneumolysin protein was diluted serially in PBS from 2,500 ng/ml down to 19.5 ng/ml in a 96-well microtiter round-bottom plate. The erythrocyte suspension of citrated sheep blood was washed twice with PBS, and 100 μl of a 1:50 dilution was added to 100 μl of lysis buffer, containing 10 mM DTT and 0.1% bovine serum albumin. The plates were incubated at 37°C for 30 min and then centrifuged at 1,500 × *g* for 10 min at RT. Erythrocyte-free PBS and lysis buffer were used as negative controls. Experiments were performed in three independent assays, each in duplicates, and the presence or absence of erythrocyte sediment was photographically monitored.

### Endothelial Chamber Separation Cell Migration Analysis

The chamber separation cell migration analysis (CSMA) was established for determination of the speed and closure efficiency of a defined gap of 500 μm in diameter that is generated between two confluently grown endothelial cell layers. For this purpose, a three-chambered silicone inlet from ibidi^®^ was placed on a 35-mm petri dish suitable for microscopic visualization. Each chamber covers a growth area of 0.22 cm^2^ with a median width of 500 μm. The bottom of three adjacent inlet chambers was coated with 2% porcine gelatin for 1 h at 37°C. Excess of gelatin was removed by two wash steps with 0.1 M PBS (pH 7.4). Further, 70 μl of 3 × 10^5^/ml HUVEC suspension was seeded into each of the three adjacent inlet chambers and cultivated for 18 h at 37°C and 5% CO_2_ to reach confluence. Cell migration analyses were started by removing the three-chambered silicone inlet. The dish was washed twice with ECGM to remove unattached HUVEC, and cellular gap closure was analyzed microscopically using a confocal laser scanning microscope (CLSM) at different time points of cultivation. The microscope setting was adjusted using the bright-field mode of the CLSM (Leica, SP8, DMI8) with the × 20/0.75 IMM objective and a zoom factor of 0.75. For evaluation of wound healing parameters, both cell borders were microscopically visualized and snap shots were taken at three different representative sights of view. The imaged region size constantly covered 775 μm × 775 μm. For calculation of the cell-free gap area per field of view, the cell borders of the cell-free gaps were marked using the LAS X software tool. At each observation time point, the size of the remaining cell-free area was determined *via* the area determination tool of the LAS X microscope software. The values were put in relation to the size of the total cell-free area determined at time *t* = 0 h, which was normalized to 0% gap closure. The percentage of gap closure (i) was calculated according to the following formula:


$$\mathrm{gap}\mathrm{closure}\left[\%\right]=\frac{\mathrm{gap}\mathrm{area}\left(\text{t}=0\text{h}\right)-\mathrm{gap}\mathrm{area}\left(\text{t}\right)}{\text{area}\left(\text{t}=0\text{h}\right)}\times 100$$

Additionally, the average velocity of the reduction of the cell-free area within 24 h of monitoring was calculated based on the difference between the values of the remaining cell-free area between each monitored period of time of gap closure. This value is divided by 24 h, which resulted in the average rate of gap closure per hour in μm^2^/h. Assuming that the cell-free area reduces linearly over time, the following formula according to Bobadilla and colleague’s “monolayer edge velocimetry” method was applied for calculation of the average gap closure rate [*U*_w_] (ii, Bobadilla et al., 2019).

(ii)


$$\mathrm{Average}\,\mathrm{gap}\,\mathrm{closure}\,\mathrm{rate}:U_{W}\,\left[\frac{\mu \,{\text{m}}^{2}}{\text{h}}\right]=\frac{\mathrm{gap}\mathrm{area}\left(\text{t}=0\text{h}\right)-\mathrm{gap}\mathrm{area}\left(\text{t}\right)}{\text{t}}$$

To determine the impact of pneumococci on the velocity and efficiency of endothelial wound healing, wild-type serotype 35A pneumococci and a pneumolysin-deficient (Δ*ply*) derivative were added to the cell layers using a multiplicity of infection (MOI) of 10, after the three-chambered inlet was removed. The culture medium was exchanged after 1 h and additionally after 6 h of cell cultivation. The microscopic visualization and evaluation of gap closure were performed as described above. Assays were performed in replicates in three independent analyses and statistically evaluated, as described below. Of note, with the aim to prevent unspecific effects due to increasing amounts of bacteria during 24 h of CSMA, uncontrolled bacterial growth throughout the CSMA was prevented by medium exchange at specific time points during CSMA. The same experimental setup was performed with heat-inactivated pneumococci. In addition, recombinantly produced pneumolysin was employed in the CSMA. Based on the results of toxic activity against erythrocytes, CSMA experiments were performed as described above in the presence of 50, 250, 375, and 500 ng/ml pneumolysin protein.

### Immunofluorescence Staining and Confocal Microscopy

For immunofluorescence staining, HUVEC were seeded in three-chambered silicone inlets, which were placed onto gelatin-coated glass coverslips into a 24-well plate. The CSMA was performed with and without bacteria, as described above. After each time point, the cell culture medium was removed, and after two wash steps with PBS, cell growth was stopped by fixation with 3% paraformaldehyde (PFA) in PBS at 4°C o/n. Antibody-mediated fluorescent staining was performed in a dark chamber and was started by blocking for 1 h at RT with 5% bovine serum albumin (BSA). All staining steps were performed in a volume of 50 μl and were separated by intensive washing in PBS reservoirs. Probes without bacteria were incubated with Alexa 488-conjugated phalloidin (1:1,000 dilution) and with DAPI (in 1:1,000 dilution). For differential staining of attached and internalized pneumococci, the bacteria-incubated probes were additionally incubated with primary pneumococcus-specific antibodies (1:100 dilution) followed by an Alexa 488-conjugated secondary anti-rabbit antibody (1:1,000 dilution). After cell permeabilization using 0.1 Triton X-100 for 5 min, the internalized pneumococci were detected with the polyclonal pneumococcus-specific antibody (1:100 dilution) followed by incubation with an Alexa 568-conjugated secondary antibody (1:1,000 dilution). Internalized bacteria inside the endothelial cells appear in red and bacteria attached to the cell surface appear yellow due to the overlay of both secondary conjugated antibodies. Prior to microscopic visualization, the stained probes were mounted onto glass slides using a mounting medium from Dako. A microscopical visualization of the fluorescence signals of the CSMA samples was performed using the × 20/0.75 IMM objective of the CLSM (Leica, Sp8, DMI8) and a zoom factor of 0.75 to maintain a constant size of the field of view. For visualization of attached and internalized pneumococci, the CLSM mode with the × 63/0.75 oil-immersion objective was used and results are shown as representative images of the merged fluorescence channels. The scale bars are defined in each figure legend and indicated sizes of 100 μm and of 10 μm in magnified pictures. Contrast and brightness were only slightly optimized using Adobe Photoshop CS5 (version 12.0.) without changing any results. A time-lapse movie was generated using the software “Microsoft Photos” (Microsoft corporation, version: 2021.21090.10007.0). The movie is based on eight images at a speed of one image per second and is included in the Supplementary Material.

### Determination of Endothelial Cell Proliferation

The proportion of cell proliferation and cell migration during gap closure was microscopically visualized and quantified using the EdU Click assay from Carl Roth, Germany, according to the instructions of the manufacturer. In brief, CSMA was performed as described above in the presence of 10 μM 5-ethynyl-2′-desoxyuridin (EdU), which is incorporated into newly synthesized DNA of proliferation endothelial cells during cell growth. The culture medium was exchanged after 1 h and after 6 h of cultivation followed by supplementation of the required EdU substances. After each cultivation time point, migrated cell samples were stopped by fixation with 3% PFA, as described above. For detection of cell proliferation, the cells were permeabilized using 0.1% Triton X-100 and cells were incubated with 5-TAMRA-PEG3-Azide solution. Microscopic imaging using the CLSM detected red fluorescent cell nuclei at an emission peak at 579 nm. For counterstaining of migrating endothelial cells, DAPI stain was performed as described above. The proportion of proliferating cells was microscopically quantified by counting the red fluorescent and blue fluorescent cell nuclei using the × 20/0.75 IMM objective of the CLSM (Leica, Sp8, DMI8) and a zoom factor of 0.75. For comparative quantification, the number of nuclei was determined in randomly chosen sights of view within the whole gap area of 500 nm in diameter at different time points of gap closure analyses. All experiments were performed in at least four independent assays, each at least in duplicates, and the data were expressed as mean value including standard deviation.

### Flow Cultivation and Infection

For analysis of cell migration under defined shear stress conditions, the CSMA in combination with flow cultivation was established using the microfluidic system of ibidi^®^. In brief, primary HUVEC were seeded into gelatin-coated two-chambered silicone inlets (ibidi^®^), which were placed onto ibiTreat coverslips (ibidi^®^), as described above. The cells were cultivated for at least 18 h at 37°C and 5% CO_2_ to reach confluence. The silicone inlet was removed after a gentle wash step with ECGM. Immediately after that, a sticky 0.4-mm microscope slide with Luer adaptors was placed on the ibiTreat coverslip and 150 μl of ECGM was filled into the Luer adaptor reservoirs. The coverslip was pressed onto the sticky slide with a special clamp (ibidi^®^) for 1 min at 37°C. The slide was connected to a degassed perfusion set with tubings of 50 cm in length and 1.6 mm in diameter. Cell adaptation to the flow was started at 4 dyn/cm^2^ for 30 min followed by an increase to 1 dyn/cm^2^ every half an hour until the final shear stress of 10 dyn/cm^2^ was reached. The cells were cultivated in continuous flow of 10 dyn/cm^2^ for up to 48 h using a total circulating volume of 13.6 ml ECGM. Gap closure was microscopically visualized and was calculated as described above at time points 0, 6, 24, and after 48 h. The same experimental setting was applied to illustrate cell movement and gap closure by fluorescence visualization of the actin cytoskeleton and the nuclei, as described above. A schematic workflow of the combined technique using CSMA and microfluidic cultivation is shown in Figure 5A. The microfluidic slide containing the HUVEC layer with a defined cell-free area was also incubated with Serotype 35A pneumococci (WT), grown to mid log phase, which were supplemented to the circulating cell culture medium at an MOI of 10. In accordance with the infection routine of the static assays, the cell culture medium was exchanged after 1, 6, 24, and 30 h of bacterial circulation in the microfluidic system. The effect of bacteria on the speed of gap closure was determined microscopically after fixation of the microfluidic infection and immunofluorescence staining, as described above.

### Electron Microscopic Visualization

Samples were fixed in 5% formaldehyde and 2% glutaraldehyde in 0.1 M HEPES buffer (HEPES 0.1 M, 0.09 M sucrose, 10 mM CaCl_2_, 10 mM MgCl_2_, pH 6.9) at 4°C and washed twice with TE buffer, pH 7.0 (20 mM Tris–HCl, 1.0 mM EDTA), before dehydrating in a graded series of ethanol for 15 min at each step. Samples were then subjected to critical-point drying with liquid CO_2_ (CPD 300, Leica Microsystems, Wetzlar, Germany) and sputter coated with a gold–palladium film (SCD 500, Bal-Tec, Liechtenstein) before examination in a field-emission scanning-electron microscope Zeiss Merlin (Oberkochen, Germany) using the Everhart–Thornley SE detector and the SE in-lens detector at a 75:25 ratio with an acceleration voltage of 5 kV.

### Statistical Analysis

Statistical significances were analyzed by the two-tailed unpaired *T*-test with unequal variance (Welsh test, Rasch et al., 2011) or in case of equal variances with Student’s *T*-test, as noted in figure legends. Data are based on technical replicates that derived from at least three independent experimental analyses. In case of microscopic evaluation, data from at least three different sights of view were combined for mean value calculation. The graphs display mean values and calculated standard deviations. *P*-values of < 0.05 were considered to be statistically significant and were marked by asterisks or declared in figure legends.

## Results

### *Streptococcus pneumoniae* Inhibits Endothelial Cell Growth

To model the effects of pneumococcal infection on endothelial wound healing, we established a two-chambered separation cell migration assay (CSMA), which enabled real-time microscopic visualization and reproducible quantification of the pneumococcal effect on endothelial tissue gap closure. HUVEC were seeded into a chambered cell culture inlet, which after removal generates a defined gap between the adjacent confluently grown monolayers. HUVEC typically serve as a well-characterized and widely used model cell type for functional analyses of the vascular endothelium. The cell growth within the defined gap area of the CSMA samples was microscopically monitored for up to 24 h.

**FIGURE 1:**
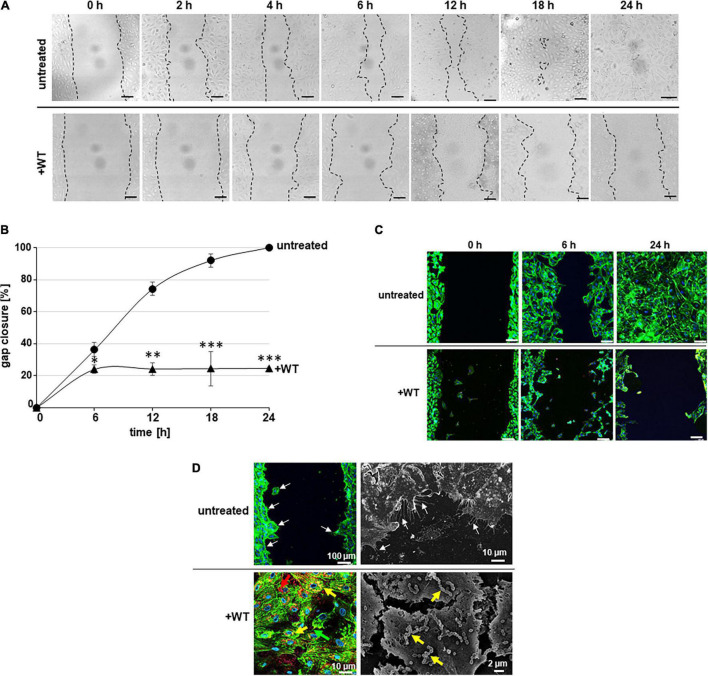
Endothelial CSMA without bacteria and in the presence of pneumococci. **(A)** Representative microscopic bright-field images of CSMA probes visualize gap closure at indicated time points (0, 1, 2, 4, 6, 12, 18, and 24 h) after removal of the inlet without bacteria (untreated) and in the presence of pneumococci (+WT) at MOI of 10. Borders of cell layers at both sides of the cell-free gap were marked with dashed lines. Scale bars represent 100 μm. **(B)** At indicated time points of cell cultivation, progress of gap closure was calculated in percent in relation to the starting point, which was defined as 0% gap closure. Black line with filled circles displays percentage of gap closure without bacteria, and black line with filled triangles visualizes gap closure in the presence of pneumococci (+WT). Standard deviation derived from three independent analyses performed in duplicates. * indicates *p* < 0.05, ^**^ indicates *p* < 0.01, and ^***^ indicates *p* < 0.001. **(C)** Endothelial cell morphology was visualized by actin cytoskeleton detection with green fluorescent phalloidin-Alexa 488 in combination with DAPI stain of nuclei at time points 0, 6, and 24 h. Representative overlay images are shown for each time point without bacteria (untreated), and in the presence of pneumococci (+WT). Images were generated using the CLSM (Leica Sp8) at 200-fold magnification. Scale bar represents 100 μm. **(D)** Fluorescence images and scanning electron microscopic illustrations of HUVEC CSMA is shown. White arrows point to cell evaginations at the growing cell borders already after 1 h of cell cultivation (untreated). In the lower panel, representative overlay fluorescence- and scanning electron microscopic images illustrate HUVEC after incubation with wild-type pneumococci. Yellow arrows point to attached pneumococci, and red arrows point to internalized pneumococci (+WT). Size of scale bars is indicated within the images.

In Figure 1A, representative images depict the fields of view, which are used for quantification of the cell-free area for CSMA without bacteria (untreated) and in the presence of wild-type pneumococci (+WT) at indicated time periods of cell cultivation. The cell borders were marked with black dashed lines using the LAS X software (Figure 1A). The bright-field images clearly visualize the reduction in the size of the cell-free area due to cell growth in untreated samples, until the gap is totally closed after 24 h (Figure 1A, untreated). The generated time-lapse movie displays closure of the cell-free gap within 24 h (Supplementary Movie). In the presence of pneumococci, overall cell growth was significantly reduced at each visualized time point and a substantial cell-free gap area was still detected after 24 h (Figure 1A, +WT). Already after 6 h of cell cultivation and longer, significant differences in gap closure between the untreated samples and the CSMA samples with bacteria were observed and provide evidence that the presence of pneumococci significantly delayed the gap closure of the HUVEC layer (Figure 1B). Quantification of the cell-free area revealed that 90% ± 5% of the gap area was closed already after 18 h of cultivation and a total gap closure (100%) was achieved after 24 h at the latest in untreated samples without bacteria (Figures 1A,B, untreated). Fluorescence staining of the actin cytoskeleton and the cell nuclei confirmed the stepwise reduction of the remaining cell-free gap area until the cell-free area was completely closed after 24 h of incubation without bacteria (Figure 1C, untreated). The cells remained attached to the gelatinated coverslip surface during the whole cultivation period, and the actin cytoskeleton stain further revealed a cobblestone-like typical cell morphology within the growing cell layer. Moreover, already 1 h after starting the CSMA cell cultivation, cells with protruded cell evaginations and extended filopodia were detected at the outer cell border directing toward the center of the gap-free area *via* fluorescence imaging and also *via* electron microscopic visualization (Figure 1D, untreated, white arrows). In contrast, after 12 h only 24 ± 1% of the gap area was closed and even 72 ± 3% of the gap still remained cell free after 24 h of incubation in the presence of pneumococci at an MOI of 10 (Figures 1B,C +WT). After differential immune fluorescence staining, microscopic magnification of a cell region within the cell layer beyond the cell-free gap displays numerous regions of cell lesions and adjacent cells generate long and thin stress fibers (Figure 1D, +WT, green arrows). This cell morphology predominantly appears in the presence of pneumococci. As shown in Figure 1D, yellow arrows point to attached pneumococci and internalized bacteria appear in red, as shown in Figure 1D (+WT). The electron microscopic magnification illustrates the attachment of pneumococci to HUVEC (yellow arrows in Figure 1D, +WT). The cells lack the typical cell morphology and appear as diffuse cell clusters with undefined cell borders.

### Endothelial Gap Closure Is Less Affected by Pneumolysin-Deficient Pneumococci

The pore-forming toxin pneumolysin is described as the main inducer of cytolytic pore formation during pneumococcus infection and in particular as substance causing damages to the brain endothelium (Zysk et al., 2001). With the aim to clarify the impact of cytotoxic effects of pneumolysin on the endothelial gap closure, a pneumolysin-deficient isogenic derivative of the serotype 35A pneumococcus strain (Δ*ply*) was included in CSMA experiments.

**FIGURE 2:**
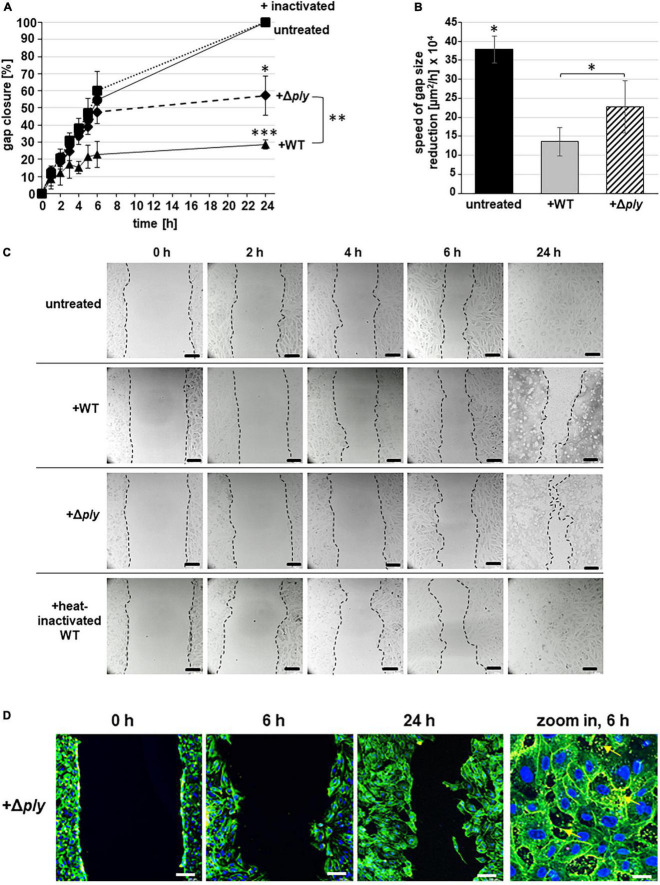
CSMA with heat-inactivated and pneumolysin-deficient pneumococci. **(A)** Percentage of gap closure is calculated at indicated time points of CSMA for up to 24 h. Data derived from experiments with pneumococci at MOI of 10 (+WT, black line with triangles), with the pneumolysin-deficient strain (+Δ*ply*, dashed line with rombi), and with the same amount of heat-inactivated wild-type pneumococci (inactivated, dotted line with squares). Gap closure of untreated HUVEC was also determined as control (black line with filled circles). All experiments were performed in three independent assays, using three-chambered inlets, and the data were expressed as mean value including standard deviation. * indicates *p* < 0.05, ^**^ indicates *p* < 0.01, and ^***^ indicates *p* < 0.001 according to two-tailed Welch’s *T*-test. **(B)** Average speed of gap size reduction (*U*_*W*_) for the whole incubation period of 24 h was calculated for untreated CSMA samples and after 24 h of incubation with wild-type bacteria (+WT) and with pneumolysin-deficient bacteria (+Δ*ply*) based on the determined cell-free gap area according to Bobadilla et al. (2019). * indicates *p* < 0.05. **(C)** Representative microscopic bright-field images of CSMA probes visualize gap closure at indicated time points after removal of the inlet without bacteria (untreated), in the presence of wild-type pneumococci (+WT), in the presence of pneumolysin-deficient pneumococci (+Δ*ply*), and in the presence of heat-inactivated wild-type pneumococci (+heat-inactivated) at MOI of 10. Cell borders were marked with dashed lines. Scale bars represent 100 μm. **(D)** Endothelial cell morphology was visualized by actin cytoskeleton detection with green fluorescent phalloidin Alexa 488 in combination with DAPI stain of nuclei at time points 0, 6, and 24 h in the presence of pneumolysin-deficient pneumococci (+Δ*ply*). Images were generated using the CLSM (Leica Sp8) at 200-fold magnification. In addition, a zoom-in magnification illustrates the attachment of Δ*ply* bacteria after differential immunofluorescence staining to HUVEC. Scale bar represents 100 μm. The bacteria appear in yellow due to the fluorescent overlay of the secondary antibodies (+Δ*ply*, zoom in 6 h, yellow arrows, scale bar represents 10 μm).

Quantification of the cell-free area confirmed that the gap closure is significantly reduced in the presence of Δ*ply* pneumococci with an MOI of 10 reaching a maximum of 57.14 ± 11.5% gap closure after 24 h of cell cultivation (Figure 2A, dashed line). This retardation in gap closure is also illustrated by representative microscopic bright-field images (Figure 2C, Δ*ply*). Without bacteria, the gap is closed after 24 h of incubation (Figure 2A, black line and Figure 2C, untreated). Compared to the lowest gap closure of 28.69 ± 2.52% after 24 h of cell cultivation in the presence of the wild-type strain (Figure 2C, WT, black line with triangles and Figure 2C, +WT), the percentage of gap closure in the presence of Δ*ply* bacteria (57.14 ± 11.5%) is rather doubled. According to the curve slope between 6 and 24 h of cell cultivation, the velocity of gap closure in the presence of Δ*ply* pneumococci is strongly reduced compared to the speed of gap closure without bacteria (Figure 2A). With the aim to assess whether metabolic active bacteria are required to cause the monitored effects on endothelial cell growth, the same amount of heat-inactivated pneumococci was employed in the CSMA. In contrast to the lack of gap closure in the presence of wild-type pneumococci, a complete gap closure is reached after 24 h of incubation in the presence of heat-inactivated pneumococci, indicating that metabolic active pneumococci are required to interfere with cell growth (Figures 2A,C, +heat-inactivated WT, dotted line). Calculation of the velocity of gap closure for the whole incubation period of 24 h according to the method of monolayer edge velocimetry revealed the highest values for the speed of gap closure of HUVEC without pneumococci (Figure 2B, untreated, Bobadilla et al., 2019). Cell growth was significantly slowed down to one-third of the speed of HUVEC without bacteria in the presence of wild-type bacteria (Figure 2B, untreated, +WT). Without the cytotoxic effects due to pneumolysin production, the velocity of gap closure was only 1.7-fold less compared to untreated HUVEC (Figure 2B, +Δ*ply*). Fluorescence microscopy revealed that the endothelial cell morphology of HUVEC with pneumolysin-deficient pneumococci is similar to untreated HUVEC (Figure 2D, +Δ*ply*). Of note, despite the high amount of Δ*ply* bacterial attachment, which is visualized in the magnified image (Figure 2D, zoom in, 6 h), the actin stain revealed no substantial changes in endothelial cell morphology. At 200-fold magnification, attached pneumococci are visualized in yellow due to the fluorescence overlay after differential immunofluorescence staining (Figure 2D, +Δ*ply*, zoom in 6 h, yellow arrows). Thus, no stress fibers and no areas of cell lesions are detected in cell layers incubated with pneumolysin-deficient pneumococci (Figure 2D, +Δ*ply*, zoom in 6 h). In sum, despite the lack of substantial changes in cell morphology, the velocity of cell growth and the efficacy of gap closure are significantly reduced in samples with Δ*ply* bacteria even after 24 h. Therefore, the impact of the pneumolysin toxin itself on endothelial gap closure is further investigated in more detail.

### Pneumolysin Inhibits Cell Proliferation During Endothelial Gap Closure

**FIGURE 3:**
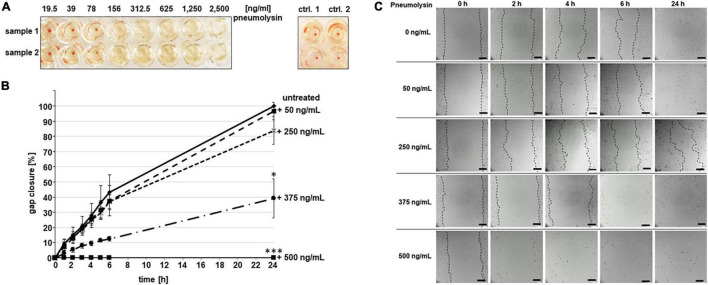
Effect of purified pneumolysin protein on endothelial cell growth. **(A)** Toxic activity of pneumolysin was assessed by standardized hemolysis assay with pneumolysin protein in serial dilution between 19.5 and up to 2,500 ng/ml. The assay was repeated three times using technical duplicates. As controls, non-incubated erythrocytes (control 1) and buffer-incubated erythrocytes (control 2) were included. **(B)** CSMA was performed in the presence of pneumolysin protein in amounts of 50 ng/ml (dashed line with squares), 250 ng/ml (dashed line with crosses), 375 ng/ml (dashed line with dots), and 500 ng/ml (black line with filled squares; placed on the zero mark of the *x*-axis due to complete cell detachment). Gap closure was monitored and quantified after indicated time points for up to 24 h. Gap closure of untreated HUVEC was also determined as control (black line with filled rombi). All experiments were performed in three independent assays using three-chambered inlets, and data were expressed as mean value including standard deviation. * indicates *p* < 0.05, and *** indicates *p* < 0.01 according to two-tailed Welch’s *T*-test. **(C)** Representative images of live cell monitoring at time points 0, 2, 4, 6, and 24 h after incubation with indicated concentrations of pneumolysin protein. Scale bar represents 100 μm.

To clarify the impact of pneumolysin-mediated cytotoxicity on the velocity and efficacy of cellular gap closure, we included pneumolysin in the CSMA. First, the activity of the purified pneumolysin protein was determined by standardized hemolysis analysis (Figure 3A). Incubation of the erythrocyte solution with a serial dilution of pneumolysin protein ranging from 2,500 ng/ml down to 19.5 ng/ml revealed that lysis of blood cells occurred in the presence of 156 ng/ml pneumolysin and higher protein amounts (Figure 3A). Based on the results of the lytic activity toward sheep erythrocytes, CSMA experiments were performed in the presence of 50, 250, 375, and 500 ng/ml pneumolysin protein (Figures 3B,C). In contrast to 100% gap closure, which is constantly reached by untreated HUVEC without toxin after 24 h, the lowest amount of 50 ng/ml pneumolysin reduced the mean value of gap closure to 96.58 ± 5.73% (Figure 3B, dashed line with squares). Only 83.73 ± 9.14% gap closure was achieved after 24 h in the presence of 250 ng/ml pneumolysin (Figure 3B, dashed line with crosses). At this toxin concentration, which already led to lysis of sheep erythrocytes, a substantial area remained cell free, although several endothelial cells remained attached to the slide surface (Figure 3C). A significantly less gap closure was monitored after HUVEC incubation with 375 ng/ml pneumolysin reaching values of only 39.14 ± 12.76% gap closure after 24 h (Figure 3B, dashed line with dots). Within the first 4 h, a maximal retardation in cell growth is detected in these samples, leaving the size of the cell-free area rather unchanged (Figure 3C). After longer incubation times, massive cell detachment occurred in the presence of 375 ng/ml pneumolysin, which effectively prevents any further cell growth (Figure 3C). Incubation of HUVEC with 500 ng/ml pneumolysin instantly induced massive cell damage and cell detachment and therefore gap closure remained unattainable (Figures 3A,B). Representative bright-field visualization confirmed the increasing cell damage and loss of cells already after 2 h of incubation with 500 ng/ml pneumolysin (Figure 3C). These data confirmed the substantial effect of the cytotoxin pneumolysin to gap closure of wounded endothelial cell layers.

### Pneumococcal Inhibition of Both Cell Proliferation and Cell Migration Contributes to Retardation of Gap Closure

**FIGURE 4:**
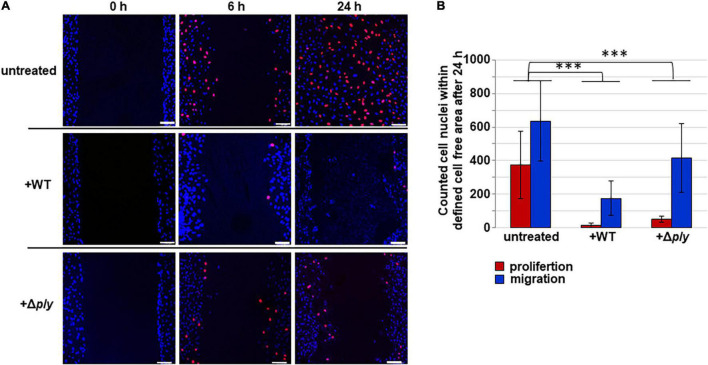
Differential microscopic evaluation of cell proliferation and cell migration. **(A)** CSMA was performed for indicated time periods without bacteria (untreated), with serotype 35 A pneumococci (+WT) at MOI of 10 and with pneumolysin-deficient derivative (+Δ*ply*) in the presence of 5-ethynyl-2′-deoxythymidine (EdU-Click), which incorporates into proliferating cell nuclei. In combination with 6-FAM-azide, nuclei of proliferating cells appear in light red, whereas migrating cells are counter-stained in blue by DAPI incubation. In CSMA samples without bacteria, gap closure was completed after 24 h of cell cultivation. In the presence of wild-type pneumococci and in the presence of pneumolysin-deficient pneumococci, the cell-free gaps are not completely closed after 24 h of cell cultivation. Representative microscopic fluorescence images are shown for each sample. Scale bar indicates 100 μm. **(B)** Proportion of proliferating cells versus migrating cells was quantified by counting the red and blue fluorescing cell nuclei in the defined gap area at indicated time points (0, 6, and 24 h). Red bars indicate cell proliferation, and blue bars indicate cell migration. Experiments were performed in three independent assays, each in triplicate, and a minimum of four different fields of view were randomly chosen for quantification of cell nuclei. Data are expressed as mean value including standard deviation. ^***^ indicates *p* < 0.001 according to two-tailed Welch’s *T*-test.

Gap closure of the cell-free area might be achieved by two different cell growth mechanisms: cell migration and cell proliferation. We aimed to decipher the proportion of cell proliferation from total gap closure by applying the EdU-Click technique in the CSMA. This technique enables to determine the proportion of proliferating cells from the total amount of migrating cells at a specific time point. Cells with DAPI-stained blue fluorescent nuclei, which were present within the defined gap after 24 h of cell cultivation, were defined and counted as migrating but not proliferating cells. The incorporation of 5-ethynyl-2′-deoxythymidine into newly synthesized DNA enabled the microscopic detection of red fluorescent nuclei of proliferating cells within the defined cell-free area at 0, 6, and 24 h. In untreated samples, the amount of proliferating cells with red fluorescent nuclei is significantly increasing in the defined gap area over time, reaching an average amount of 374 proliferating cells (Figures 4A,B, untreated, red bars). In contrast, rather no cell proliferation is displayed in the presence of pneumococci (mean of 14 cells) and only an average amount of 50 red nuclei are detected in CSMA samples incubated with Δ*ply*–pneumococci, which is rather 7.5-fold less and significantly different from untreated samples (Figures 4A,B, +WT, +Δ*ply*). A mean value of 635 migrating but not proliferating cells with blue fluorescent nuclei was counted within the defined area of the closed gap after 24 h of HUVEC cultivation (Figure 4B, untreated, blue bar). Significantly less non-proliferating cells were detected in the presence of wild-type bacteria reaching only a maximum of 175 migrating cells within the gap area. This is rather four-fold less cell migration compared to the HUVEC sample without bacteria. Interestingly, in the presence of the pneumolysin-deficient strain, a mean value of 414 non-proliferating cells migrated into the gap area, which is significantly more than two-fold cell migration compared to the migration in the presence of the wild-type bacteria (Figure 4B, blue bars). In all samples with and without bacteria, the amount of cell proliferation was lower than the amount of cell migration. The data provide strong evidence that the incubation with pneumococci significantly affects both cell migration and cell proliferation of HUVEC, showing stronger effects on cell proliferation even in the absence of the cytotoxic effects of pneumolysin.

**FIGURE 5:**
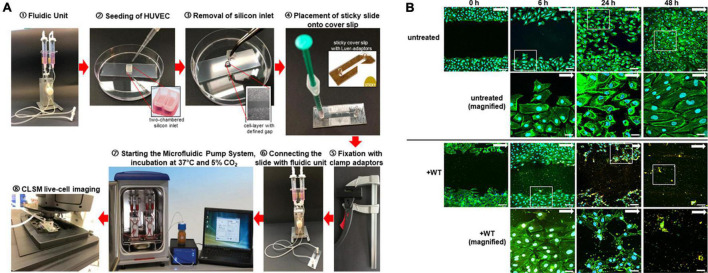
Workflow and fluorescence images of chamber separation migration analyses under defined shear stress conditions. **(A)** Workflow. Two pump reservoirs of a fluidic unit are filled with cell culture medium for cell cultivation in flow ➀. For cell migration analysis, the cells are seeded into the two chambers of a gelatin-coated silicone inlet placed on a microscope slide ➁. After cell cultivation, the silicone inlet is removed leaving a defined spatial distance of 500 μm between the two cell layers ➂. The microscope slide is covered with a “sticky coverslip” leaving a groove for perfusion with cell culture medium ➃. After the sticky coverslip is fixed with clamping tongs to ensure tight coverage over the whole time of flow cultivation ➄, the connection of the slide with the perfusion was set ➅. According to the chosen fluidic parameters, the defined continuous and unidirectional microfluidic perfusion is started ➆. Cell migration is microscopically visualized using the CLSM Sp8 at different time points ➇. **(B)** Representative microscopic fluorescence images of HUVEC gap closure at time points 0, 6, 24, and 48 h of microfluidic cultivation at 10 dyn/cm^2^. After indicated incubation times, CSMA samples without bacteria (untreated) and with pneumococci (+WT) were stained using green fluorescent phalloidin Alexa-488 in combination with DAPI for detection of cellular nuclei in blue. In case of bacterial incubation, differential immuno fluorescence staining visualizes attached bacteria in yellow. White arrows mark the direction of flow, and white squares encircle the region of magnification taken at 630-fold magnification each shown in the image below. Images were generated using the CLSM (Leica Sp8) at 200-fold magnification. Scale bar represents 100 and 10 μm, respectively, in the magnifications.

### Endothelial Cell Growth in the Presence of Physiological Shear Stress

Flow-dependent vascular shear stress has substantial impact on cell proliferation and migration and is crucial for the maintenance of endothelial barrier integrity (Rousseau et al., 2000; Chistiakov et al., 2017). With the aim to establish a model system, which enables the microscopic visualization and quantification of the effect of pneumococcal virulence factors on endothelial migration and wound healing under physiological shear stress conditions, we combined the CSMA with the microfluidic pump system. As depicted in Figure 5A, the CSMA was initiated as described above by using two-chambered silicone inlets for standardized generation of a defined cell-free gap between two confluently grown endothelial cell layers. After covering the CSMA sample with a sticky slide, the whole slide was air free connected with the microfluidic pump system *via* Luer adaptors. The cells were further cultivated under defined shear stress of up to 10 dyn/cm^2^. The slides are suitable for microscopic live cell imaging and for long-term flow cultivation of endothelial cells. Cell growth and gap closure were determined by microscopic monitoring. Flow speed and shear stress parameters are set and controlled *via* a special Pump software and mimicked the desired blood vessel situation.

Representative fluorescence images of endothelial gap closure in continuous and defined flow of 10 dyn/cm^2^ indicated that HUVEC require 48 h to close the cell-free area, which doubled the time required for gap closure without flow (Figure 5B, untreated). Staining of the actin cytoskeleton with green fluorescent phalloidin illustrates an increasing cell density reaching a tightly packed cell layer after 24 h. Instead of the complete gap closure, which is reached by untreated HUVEC after 24 h of flow incubation (Figure 5B, untreated), the gap is not closed in the presence of pneumococci even after 48 h of constant flow incubation. In fact, the size of the cell-free area is substantially increased in the presence of pneumococci (Figure 5B, +WT, 24 h). The visual evaluation of the microscopic images indicates a reduction in the cell-free area after 24 h of incubation in flow even in the presence of wild-type pneumococci (Figure 5B, untreated and +WT). Moreover, the magnified images visualize representative cells of the cell area within the white squares, which are oriented in line with the flow direction (Figure 5B, untreated and +WT). After 6 h of flow incubation with circulating pneumococci, the cells appear in typical morphological shape, whereas after 24 h of flow cultivation, the phalloidin stain of the cellular actin cytoskeleton visualizes damaged cells at the wound borders, many lesions in the cell layer, and stress fibers (Figure 5B, +WT, 24 h). Finally, a massive cell detachment is microscopically detected after 48 h of bacterial incubation (Figure 5B, +WT, 48 h). These results demonstrate the substantial impact of pneumococci on endothelial growth under defined shear stress.

In addition to the static cell migration analyses, the combination of endothelial cell migration slides with a microfluidic pump system also enabled the simulation of tissue regeneration under physiological vascular shear stress of defined values. The combined technique serves as suitable model to analyze the effect of bacterial adherence on activated and damaged endothelial cells during sepsis in blood circulation.

## Discussion

With the aim to decipher the effect of pneumococcal infection on endothelial cell migration, we established with the CSMA a wound healing assay, which is suitable for the analyses of HUVEC migration in the presence of pneumococci in frame of a standardized cell culture infection protocol, initially without any simulation of flow conditions. In contrast to the classical method of mechanical scratching of cell wounds into a formerly grown cell layer, we cultivate the cells in specialized silicone inlets. These inlets generate a defined gap of 500 μm in diameter between the cell layers growing in adjacent inlet chambers. This assay comprises all advantages of a classical scratch assay such as stimulation of cell migration as individual cells or groupwise in direction of the cell-free area, thereby mimicking the behavior of these cells during migration *in vivo* (Liang et al., 2007). In addition, the CSMA is also compatible with microscopy including live cell imaging, allowing analysis of cell morphology and intracellular signaling events. In contrast to the mechanical scratching assay, only small amounts of primary cells are required for cell cultivation within the CSMA inlet chambers, and only a minor amount of biochemicals are required for example for immunofluorescence staining. The use of multichambered silicone inlets enables a direct comparison of several different samples within one assay. Microscopic visualization results of HUVEC in the CSMA system confirmed the successful stimulation of endothelial migration after removal of the silicone chamber inlet, which provides the external stimuli for directed cell migration.

Depending on the tissue environment *in vivo* and on the extent of the vessel damage, endothelial cells are known to migrate individually, as chains or groupwise in sheets (Michaelis, 2014). Collectively migrating cells form a front–rear symmetry, which divides the cell group in single “leader cells,” also called tip cells, and in “followers” (Vitorino and Meyer, 2008). This concerted endothelial cell mobility constitutes an important prerequisite to achieve vascular regeneration and barrier integrity (Reinhart-King, 2008). For an effective migration within a vascular cell environment, a persistent movement toward a specific direction is required, which begins with the polarization of each individual cell and includes the steady formation of cytoskeleton-dependent protrusions (Michaelis, 2014). In line with this, already 1 h after removal of the inlet, our cell imaging reveals the presence of cell protrusions along the damaged cell border and also the presence of single leader cells heading toward the center of the cell-free gap. Without infection, the cell-free area was completely closed after 24 h. In contrast, incubation of HUVEC with wild-type pneumococci leads to significant inhibition of both endothelial cell migration and endothelial cell proliferation. Moreover, in contrast to the regular cobblestone-like appearance of the cell shape without bacteria, the actin cytoskeleton staining clearly visualized the formation of long and thin stress fibers in the presence of pneumococci. In addition, cell debris and cell-free lesions were monitored within the formerly grown cell layer and significantly less cell proliferation was quantified. Thus, in none of our CSMA gap closure was achieved in assays with wild-type pneumococci. These results correspond with reports describing loss of endothelial barrier permeability due to bacterial infections.

An increase in interstitial leakage and vascular barrier permeability is caused by cell apoptosis and by cell damage, which is substantially mediated by cytotoxic and cytolytic effects of bacterial toxins such as pneumococcal pneumolysin (Grandel and Grimminger, 2003; Opal and van der Poll, 2015; Colbert and Schmidt, 2016). Pneumococci ubiquitously release this cytotoxin *via* enzyme-mediated autolysis, competence-induced autolysis, and antibiotic-mediated lysis (Paton et al., 1993; Spreer et al., 2003; Guiral et al., 2005). Pneumolysin binds to cholesterol of host cells, oligomerizes, and induces membrane pores leading to cell lysis (Tilley et al., 2005; Cassidy and O’Riordan, 2013). The general cytotoxicity of pneumolysin is well documented, and this toxin has been shown to be crucial for pneumococcal virulence and invasion *in vivo* (Canvin et al., 1995; Zysk et al., 2001; Braun et al., 2002; Orihuela et al., 2004; Schmeck et al., 2004). In a former study, the effect of pneumolysin on primary endothelial cells under static conditions was determined by quantification of lactate dehydrogenase within the cell culture supernatant. In response to incubation with 3 ng/ml pneumolysin for 4.5 h, pulmonary endothelial cells released a substantial amount of lactate dehydrogenase into the cell culture supernatant, which is used as a reliable indicator for cytotoxic cell death (Luttge et al., 2012, please refer to Supplementary Data 1A). In line with our data, cytotoxic effects causing cell damage and lesion formation have been described for other streptococcal cholesterol-depending pore-forming toxins, such as suilysin of *Streptococcus suis* (Meng et al., 2016). In addition to its lytic activity, pneumolysin also displays several distinct effects on host cells even in sublytic concentrations such as the induction of myocardial injury, the activation of proinflammatory immune cells, and the destruction of platelets (Houldsworth et al., 1994; Cockeran et al., 2001; Cassidy and O’Riordan, 2013; Alhamdi et al., 2015; Jahn et al., 2020). Moreover, in sublytic concentrations, pneumolysin also interacts with the mannose receptor C type 1 (MRC-1) on specific immune cells, which compromises the epithelial barrier integrity in lung tissues (Subramanian et al., 2020). Moreover, it had been demonstrated that pneumolysin induces DNA damage and cell cycle arrest in alveolar epithelial cells (Rai et al., 2016). This genotoxic effect of pneumolysin might also explain the significantly less cell proliferation, which we monitored in our endothelial CSMA. In conclusion, the cytotoxic and cytolytic effects of pneumolysin might explain the microscopically monitored cell damage and the loss of endothelial gap closure in the presence of wild-type pneumococci. Interestingly, despite the lack of the pneumolysin-mediated cytotoxic effects, a significant retardation of gap closure is also monitored in the presence of pneumolysin-deficient pneumococci. Together with the observation that only metabolically active bacteria inhibit endothelial gap closure, whereas rather no inhibition is monitored by heat-inactivated bacteria, lack of gap closure in the presence of pneumolysin-deficient bacteria provides evidence that additional pneumococcal virulence factors might substantially interfere with endothelial cell migration and cell proliferation. The identification of these factors is planned for our ongoing studies.

Endothelial cell surfaces are exposed to the shear stress of the blood flow. Depending on the diameter of the vessel, flow speed, and viscosity, average shear stress values between 1 and 18 dyn/cm^2^ are described (Reneman et al., 2006). So far, most of the results published to date on the interaction of pneumococci with endothelial cells had been generated by using static, two-dimensional infection models, in which no blood flow effects were taken into account (Bergmann et al., 2009; Zakrzewicz et al., 2016). However, according to recently published cell biology-oriented studies, the effect of shear stress generated by the hemodynamic forces of the bloodstream on vascular cells is significantly modulating cellular proliferation and migration (Rousseau et al., 2000; Chistiakov et al., 2017; Shih et al., 2019). Only recently, it became evident that shear forces are sensed by special protein complexes named endothelial mechanosomes, which mediate a shear stress-dependent remodeling of the intracellular actin cytoskeleton and are therefore directly involved in processes of cell proliferation and migration of endothelial cells (Conway and Schwartz, 2012; Chistiakov et al., 2017). Therefore, the simulation of the physiological shear stress conditions of the blood stream is required, if mechanoresponsive factors shall be included in the characterization of the pneumococcal effect on endothelial cell migration. Based on our experience on the microfluidic system of ibidi^®^ (Jagau et al., 2019a,b), we combined the CSMA technique with the microfluidic system to enable reproducible and reliable CSMA under defined continuous shear stress and microscopic live cell analyses. In addition to the broad technical analysis options, flow cultivation of endothelial cells is known to effectively promote cell differentiation, which shifts cell migration analyses further into a more physiological condition. We established a shear stress-adaptation protocol for HUVEC to keep the already confluently grown cell layer within the two adjacent silicone chambers attached during the flow incubation. We stepwise accelerated the shear stress from 3 dyn/cm^2^ up to a continuous flow of 10 dyn/cm^2^, which is described as typical shear stress present in blood vessels of an average size. A microscopic evaluation of our CSMA samples in flow confirmed the attachment of two cell populations throughout the CSMA and orientation of endothelial cells in direction of the flow, which indicates a successful adaptation to the applied shear stress. Indeed, endothelial gap closure was achieved by cell migration and cell proliferation even in our CSMA under flow. Hence, gap closure of HUVEC in flow was reached after 48 h of flow cultivation due to the cellular adaptation process to the applied shear stress. In line with the CSMA results under static conditions, no endothelial gap closure was monitored after 48 h in the presence of pneumococci. These results provide evidence that the combined microfluidic CSMA technique is suitable for analyses of endothelial cell migration at defined shear stress. Moreover, this technique offers a broad variety of analysis options for the characterization of pathogenicity mechanisms in simulated blood stream conditions. The development of a combined endothelial cell migration analysis technique with a reliable microfluidic system generating a defined shear stress condition throughout bacterial incubation provides a powerful technique to characterize pathophysiological processes in real time.

## Data Availability Statement

The original contributions presented in the study are included in the article/Supplementary Material, further inquiries can be directed to the corresponding author.

## Author Contributions

AK, BC, and IR established, optimized, and performed wound healing analysis. SB designed the experiments and wrote the manuscript. SH provided the pneumolysin and pneumolysin-deficient mutants and edited the manuscript. MF and MS critically reviewed the manuscript. MM performed electron microscopic studies. All authors contributed to the article and approved the submitted version.

## Conflict of Interest

The authors declare that the research was conducted in the absence of any commercial or financial relationships that could be construed as a potential conflict of interest.

## Publisher’s Note

All claims expressed in this article are solely those of the authors and do not necessarily represent those of their affiliated organizations, or those of the publisher, the editors and the reviewers. Any product that may be evaluated in this article, or claim that may be made by its manufacturer, is not guaranteed or endorsed by the publisher.

## Supplementary Material

Supplementary Video 1 Time lapse movie.

## References

[B1] Abdullahi O. Nyiro J. Lewa P. Slack M. Scott J. A. (2008). The descriptive epidemiology of *Streptococcus pneumoniae* and *Haemophilus influenzae* nasopharyngeal carriage in children and adults in Kilifi district. *Kenya Pediatr. Infect. Dis. J.* 27 59–64. 10.1097/INF.0b013e31814da70c 18162940 PMC2382474

[B2] Alhamdi Y. D. R. Neill S. T. Abrams H. A. Malak R. Yahya R. Barrett-Jolley G. (2015). Circulating Pneumolysin Is a Potent Inducer of Cardiac Injury during Pneumococcal Infection. *PLoS Pathog.* 11:e1004836. 10.1371/journal.ppat.1004836 25973949 PMC4431880

[B3] Attali C. Durmort C. Vernet T. Di Guilmi A. M. (2008). The interaction of *Streptococcus pneumoniae* with plasmin mediates transmigration across endothelial and epithelial monolayers by intercellular junction cleavage. *Infect. Immun.* 76 5350–5356. 10.1128/IAI.00184-08 18725422 PMC2573366

[B4] Benton K. A. Paton J. C. Briles D. E. (1997). The hemolytic and complement-activating properties of pneumolysin do not contribute individually to virulence in a pneumococcal bacteremia model. *Microb. Pathog.* 23 201–209. 10.1006/mpat.1997.0150 9344781

[B5] Bergmann S. Hammerschmidt S. (2006). Versatility of pneumococcal surface proteins. *Microbiology* 152 295–303. 10.1099/mic.0.28610-0 16436417

[B6] Bergmann S. Hammerschmidt S. (2007). Fibrinolysis and host response in bacterial infections. *Thromb. Haemost.* 98, 512–520. 17849039

[B7] Bergmann S. Lang A. Rohde M. Agarwal V. Rennemeier C. Grashoff C. (2009). Integrin-linked kinase is required for vitronectin-mediated internalization of *Streptococcus pneumoniae* by host cells. *J. Cell Sci.* 122 256–267. 10.1242/jcs.035600 19118218

[B8] Bergmann S. Rohde M. Chhatwal G. S. Hammerschmidt S. (2001). alpha-Enolase of *Streptococcus pneumoniae* is a plasmin(ogen)-binding protein displayed on the bacterial cell surface. *Mol. Microbiol.* 40 1273–1287. 10.1046/j.1365-2958.2001.02448.x 11442827

[B9] Bergmann S. Rohde M. Preissner K. T. Hammerschmidt S. (2005). The nine residue plasminogen-binding motif of the pneumococcal enolase is the major cofactor of plasmin-mediated degradation of extracellular matrix, dissolution of fibrin and transmigration. *Thromb. Haemost.* 94 304–311. 10.1160/TH05-05-0369 16113819

[B10] Bergmann S. Schönen H. Hammerschmidt S. (2013). The interaction between bacterial enolase and plasminogen promotes adherence of *Streptococcus pneumoniae* to epithelial and endothelial cells. *Int. J. Med. Microbiol.* 303, 452–462. 10.1016/j.ijmm.2013.06.002 23906818

[B11] Bergmann S. Wild D. Diekmann O. Frank R. Bracht D. Chhatwal G. S. (2003). Identification of a novel plasmin(ogen)-binding motif in surface displayed alpha-enolase of *Streptococcus pneumoniae*. *Mol. Microbiol.* 49 411–423. 10.1046/j.1365-2958.2003.03557.x 12828639

[B12] Bobadilla A. V. P. Arevalo J. Sarro E. Byrne H. M. Maini P. K. Carraro T. (2019). *In vitro* cell migration quantification method for scratch assays. *J. R. Soc. Interface* 16:20180709. 10.1098/rsif.2018.0709 PMC6408363 30958186

[B13] Bogaert D. De Groot R. Hermans P. W. (2004). *Streptococcus pneumoniae* colonisation: the key to pneumococcal disease. *Lancet Infect. Dis.* 4 144–154. 10.1016/S1473-3099(04)00938-7 14998500

[B14] Braun J. S. Sublett D. Freyer Mitchell T. J. Cleveland J. L. Tuomanen J. E. Weber J. R. (2002). Pneumococcal pneumolysin and H(2)O(2) mediate brain cell apoptosis during meningitis. *J. Clin. Invest.* 109 19–27. 10.1172/JCI12035 11781347 PMC150815

[B15] Broughton G. II Janis J. E. Attinger C. E. (2006). Wound healing: an overview. *Plast. Reconstr. Surg.* 117 1e–S–32e–S. 10.1097/01.prs.0000222562.60260.f9 16801750

[B16] Canvin J. R. Marvin A. P. Sivakumaran M. Paton J. C. Boulnois G. J. Andrew P. W. (1995). The role of pneumolysin and autolysin in the pathology of pneumonia and septicemia in mice infected with a type 2 pneumococcus. *J. Infect. Dis.* 172 119–123. 10.1111/j.1365-2249.2004.02611.x 7797901

[B17] Cassidy S. K. O’Riordan M. X. (2013). More than a pore: the cellular response to cholesterol-dependent cytolysins. *Toxins* 5 618–636. 10.3390/toxins5040618 23584137 PMC3705283

[B18] Chistiakov D. A. Orekhov A. N. Bobryshev Y. V. (2017). Effects of shear stress on endothelial cells: go with the flow. *Acta Physiol.* 219 382–408. 10.1111/apha.12725 27246807

[B19] Cockeran R. Theron A. J. Steel H. C. Matlola N. M. Mitchell T. J. Feldman C. (2001). Proinflammatory interactions of pneumolysin with human neutrophils. *J. Infect. Dis.* 183, 604–611. 10.1086/318536 11170986

[B20] Colbert J. F. Schmidt E. P. (2016). Endothelial and Microcirculatory Function and Dysfunction in Sepsis. *Clin. Chest Med.* 37 263–275. 10.1016/j.ccm.2016.01.009 27229643 PMC4884305

[B21] Conway D. Schwartz M. A. (2012). Lessons from the endothelial junctional mechanosensory complex. *F1000 Biol. Rep.* 4 1. 10.3410/B4-1 22238515 PMC3251317

[B22] Grandel U. Grimminger F. (2003). Endothelial responses to bacterial toxins in sepsis. *Crit. Rev. Immunol.* 23 267–299. 10.1615/critrevimmunol.v23.i4.20 14700271

[B23] Guiral S. Mitchell T. J. Martin B. Claverys J. P. (2005). Competence-programmed predation of noncompetent cells in the human pathogen *Streptococcus pneumoniae*: genetic requirements. *Proc. Natl. Acad. Sci. U.S.A.* 102 8710–8715. 10.1073/pnas.0500879102 15928084 PMC1150823

[B24] Henriques-Normark B. Tuomanen E. I. (2013). The pneumococcus: epidemiology, microbiology, and pathogenesis. *Cold Spring Harb. Perspect. Med.* 3:a010215. 10.1101/cshperspect.a010215 23818515 PMC3685878

[B25] Houldsworth S. Andrew P. W. Mitchell T. J. (1994). Pneumolysin stimulates production of tumor necrosis factor alpha and interleukin-1 beta by human mononuclear phagocytes. *Infect. Immun.* 62 1501–1503. 10.1128/iai.62.4.1501-1503.1994 8132361 PMC186313

[B26] Ilina O. Friedl P. (2009). Mechanisms of collective cell migration at a glance. *J. Cell Sci.* 122 3203–3208. 10.1242/jcs.036525 19726629

[B27] Imohl M. Moller J. Reinert R. R. Perniciaro S. van der Linden M. Aktas O. (2015). Pneumococcal meningitis and vaccine effects in the era of conjugate vaccination: results of 20 years of nationwide surveillance in Germany. *BMC Infect. Dis.* 15:61. 10.1186/s12879-015-0787-1 25885764 PMC4335684

[B28] Iovino F. Molema G. Bijlsma J. J. (2014a). Platelet endothelial cell adhesion molecule-1, a putative receptor for the adhesion of *Streptococcus pneumoniae* to the vascular endothelium of the blood-brain barrier. *Infect. Immun.* 82 3555–3566. 10.1128/IAI.00046-14 24914219 PMC4187830

[B29] Iovino F. Molema G. Bijlsma J. J. (2014b). *Streptococcus pneumoniae* Interacts with pIgR expressed by the brain microvascular endothelium but does not co-localize with PAF receptor. *PLoS One* 9:e97914. 10.1371/journal.pone.0097914 24841255 PMC4026408

[B30] Iovino F. Seinen J. Henriques-Normark B. van Dijl J. M. (2016). How Does *Streptococcus pneumoniae* Invade the Brain? *Trends Microbiol.* 24 307–315. 10.1016/j.tim.2015.12.012 26804733

[B31] Jagau H. Behrens I. K. Lahme K. Lorz G. Koster R. W. Schneppenheim R. (2019a). Von Willebrand Factor Mediates Pneumococcal Aggregation and Adhesion in Blood Flow. *Front. Microbiol.* 10:511. 10.3389/fmicb.2019.00511 30972039 PMC6443961

[B32] Jagau H. Behrens I. K. Steinert M. Bergmann S. (2019b). Pneumococcus Infection of Primary Human Endothelial Cells in Constant Flow. *J. Vis. Exp.* 152:e60323. 10.3791/60323 31736484

[B33] Jahn K. Handtke S. Palankar R. Weißmüller S. Nouailles G. Kohler T. P. (2020). Pneumolysin induces platelet destruction, not platelet activation, which can be prevented by immunoglobulin preparations *in vitro*. *Blood Adv.* 4 6315–6326. 33351126 10.1182/bloodadvances.2020002372 PMC7756997

[B34] Joffre J. Hellman J. Ince C. Ait-Oufella H. (2020). Endothelial Responses in Sepsis. *Am. J. Respir. Crit. Care Med.* 202 361–370. 10.1164/rccm.201910-1911TR 32101446

[B35] Jonkman J. E. Cathcart J. A. Xu F. Bartolini M. E. Amon J. E. Stevens K. M. (2014). An introduction to the wound healing assay using live-cell microscopy. *Cell Adh. Migr.* 8 440–451. 10.4161/cam.36224 25482647 PMC5154238

[B36] Kadioglu A. Weiser J. N. Paton J. C. Andrew P. W. (2008). The role of *Streptococcus pneumoniae* virulence factors in host respiratory colonization and disease. *Nat. Rev. Microbiol.* 6 288–301. 10.1038/nrmicro1871 18340341

[B37] Liang C.-C. Park A. Y. Guan J.-L. (2007). *In vitro* scratch assay: a convenient and inexpensive method for analysis of cell migration in vitro. *Nat. Prot.* 2 329–333. 10.1038/nprot.2007.30 17406593

[B38] Loughran A. J. Orihuela C. J. Tuomanen E. I. (2019). *Streptococcus pneumoniae*: invasion and Inflammation. *Microbiol. Spectr.* 7:2. 10.1128/microbiolspec.GPP3-0004-2018 30873934 PMC6422050

[B39] Luttge M. Fulde M. Talay S. R. Nerlich A. Rohde M. Preissner K. T. (2012). *Streptococcus pneumoniae* induces exocytosis of Weibel-Palade bodies in pulmonary endothelial cells. *Cell Microbiol.* 14 210–225. 10.1111/j.1462-5822.2011.01712.x 21999205

[B40] Marriott H. M. Mitchell T. J. Dockrell D. H. (2008). Pneumolysin: a double-edged sword during the host-pathogen interaction. *Curr. Mol. Med.* 8 497–509. 10.2174/156652408785747924 18781957

[B41] Meng F. Wu N. H. Seitz M. Herrler G. Valentin-Weigand P. (2016). Efficient suilysin-mediated invasion and apoptosis in porcine respiratory epithelial cells after streptococcal infection under air-liquid interface conditions. *Sci. Rep.* 6:26748. 10.1038/srep26748 27229328 PMC4882623

[B42] Michaelis U. R. (2014). Mechanism of endothelial cell migration. *Cell Mol. Life Sci.* 71 4131–4148. 10.1007/s00018-014-1678-0 25038776 PMC11113960

[B43] Mitchell T. J. Dalziel C. E. (2014). The biology of pneumolysin. *Subcell. Biochem.* 80 145–160. 10.1007/978-94-017-8881-6_8 24798011

[B44] Opal S. M. van der Poll T. (2015). Endothelial barrier dysfunction in septic shock. *J. Intern. Med.* 277 277–293. 10.1111/joim.12331 25418337

[B45] Orihuela C. J. Gao G. Francis K. P. Yu J. Tuomanen E. I. (2004). Tissue-specific contributions of pneumococcal virulence factors to pathogenesis. *J. Infect. Dis.* 190 1661–1669. 10.1086/424596 15478073

[B46] Paton J. C. Andrew P. W. Boulnois G. J. Mitchell T. J. (1993). Molecular analysis of the pathogenicity of *Streptococcus pneumoniae*: the role of pneumococcal proteins. *Annu. Rev. Microbiol.* 47 89–115. 10.1146/annurev.mi.47.100193.000513 7903033

[B47] Peter A. Fatykhova D. Kershaw O. Gruber A. D. Rueckert J. Neudecker J. (2017). Localization and pneumococcal alteration of junction proteins in the human alveolar-capillary compartment. *Histochem. Cell Biol.* 147 707–719. 10.1007/s00418-017-1551-y 28247028

[B48] Pober J. S. Sessa W. C. (2007). Evolving functions of endothelial cells in inflammation. *Nat. Rev. Immunol.* 7 803–815. 10.1038/nri2171 17893694

[B49] Pracht D. Elm C. Gerber J. Bergmann S. Rohde M. Seiler M. (2005). PavA of *Streptococcus pneumoniae* modulates adherence, invasion, and meningeal inflammation. *Infect. Immun.* 73 2680–2689. 10.1128/IAI.73.5.2680-2689.2005 15845469 PMC1087317

[B50] Rai P. He F. Kwang J. Engelward B. P. Chow V. T. (2016). Pneumococcal Pneumolysin Induces DNA Damage and Cell Cycle Arrest. *Sci. Rep.* 6:22972. 10.1038/srep22972 27026501 PMC4812240

[B51] Rasch D. Kubinger K. D. Moder K. (2011). The two-sample t test: pre-testing its assumptions does not pay off. *Stat. Papers* 52 219–231. 10.1007/s00362-009-0224-x

[B52] Reinhart-King C. A. (2008). Endothelial cell adhesion and migration. *Meth. Enzymol.* 443 45–64. 10.1016/S0076-6879(08)02003-X 18772010

[B53] Reneman R. S. Arts T. Hoeks A. P. (2006). Wall shear stress–an important determinant of endothelial cell function and structure–in the arterial system in vivo. Discrepancies with theory. *J. Vasc. Res.* 43 251–269. 10.1159/000091648 16491020

[B54] Rousseau S. Houle F. Huot J. (2000). Integrating the VEGF signals leading to actin-based motility in vascular endothelial cells. *Trends Cardiovasc. Med.* 10 321–327. 10.1016/s1050-1738(01)00072-x 11369257

[B55] Schmeck B. Gross R. N’Guessan P. D. Hocke A. C. Hammerschmidt S. Mitchell T. J. (2004). *Streptococcus pneumoniae*-induced caspase 6-dependent apoptosis in lung epithelium. *Infect. Immun.* 72 4940–4947. 10.1128/IAI.72.9.4940-4947.2004 15321985 PMC517413

[B56] Shih H. C. Lee T. A. Wu H. M. Ko P. L. Liao W. H. Tung Y. C. (2019). Microfluidic Collective Cell Migration Assay for Study of Endothelial Cell Proliferation and Migration under Combinations of Oxygen Gradients, Tensions, and Drug Treatments. *Sci. Rep.* 9:8234. 10.1038/s41598-019-44594-5 PMC6546762 31160651

[B57] Singer A. J. Clark R. A. (1999). Cutaneous wound healing. *N. Engl. J. Med.* 341 738–746. 10471461 10.1056/NEJM199909023411006

[B58] Spreer A. Kerstan H. Bottcher T. Gerber J. Siemer A. Zysk G. (2003). Reduced release of pneumolysin by *Streptococcus pneumoniae* in vitro and in vivo after treatment with nonbacteriolytic antibiotics in comparison to ceftriaxone. *Antimicrob. Agents Chemother.* 47 2649–2654. 10.1128/AAC.47.8.2649-2654.2003 12878534 PMC166091

[B59] Subramanian K. Iovino F. Tsikourkitoudi V. Merkl P. Ahmde S. Berry S. B. (2020). Mannose receptor-derived peptides neutralize pore-forming toxins and reduce inflammation and development of pneumococcal disease. *EMBO Mol. Med.* 12:e12695. 10.15252/emmm.202012695 32985105 PMC7645366

[B60] Tasaka S. Qin L. Saijo A. Albelda S. M. DeLisser H. M. Doerschuk C. M. (2003). Platelet endothelial cell adhesion molecule-1 in neutrophil emigration during acute bacterial pneumonia in mice and rats. *Am. J. Respir. Crit. Care Med.* 167 164–170. 10.1164/rccm.2202011 12524254

[B61] Tilley S. J. Orlova E. V. Gilbert R. J. Andrew P. W. Saibil H. R. (2005). Structural basis of pore formation by the bacterial toxin pneumolysin. *Cell* 121 247–256. 10.1016/j.cell.2005.02.033 15851031

[B62] Ting L. H. Jahn J. R. Jung J. I. Shuman B. R. Feghhi S. Han S. J. (2012). Flow mechanotransduction regulates traction forces, intercellular forces, and adherens junctions. *Am. J. Physiol. Heart Circ. Physiol.* 302 H2220–H2229. 10.1152/ajpheart.00975.2011 22447948 PMC3378295

[B63] Tzima E. Irani-Tehrani M. Kiosses W. B. Dejana E. Schultz D. A. Engelhardt B. (2005). A mechanosensory complex that mediates the endothelial cell response to fluid shear stress. *Nature* 437 426–431. 10.1038/nature03952 16163360

[B64] Valles J. Diaz E. Martin-Loeches I. Bacelar N. Saludes P. Lema J. (2016). Evolution over a 15-year period of the clinical characteristics and outcomes of critically ill patients with severe community-acquired pneumonia. *Med. Intensiva* 40 238–245. 10.1016/j.medin.2015.07.005 26391738

[B65] Van der Flier M. Stockhammer G. Vonk G. J. Nikkels P. G. van Diemen-Steenvoorde R. A. van der Vlist G. J. (2001). Vascular endothelial growth factor in bacterial meningitis: detection in cerebrospinal fluid and localization in postmortem brain. *J. Infect. Dis.* 183 149–153. 10.1086/317643 11106541

[B66] Vedula S. R. Ravasio A. Lim C. T. Ladoux B. (2013). Collective cell migration: a mechanistic perspective. *Physiology* 28 370–379. 10.1152/physiol.00033.2013 24186932

[B67] Vitorino P. Meyer T. (2008). Modular control of endothelial sheet migration. *Genes Dev.* 22 3268–3281. 10.1101/gad.1725808 19056882 PMC2600767

[B68] Witzenrath M. Gutbier B. Hocke A. C. Schmeck B. Hippenstiel S. Berger K. (2006). Role of pneumolysin for the development of acute lung injury in pneumococcal pneumonia. *Crit. Care Med.* 34 1947–1954. 10.1097/01.CCM.0000220496.48295.A9 16715037

[B69] World Health Organization. (2017). *Streptococcus pneumoniae.* Available online at: https://www.who.int/news/item/27-02-2017-who-publishes-list-of-bacteria-for-which-new-antibiotics-are-urgently-needed (Accessed on Aug 2021)

[B70] Zakrzewicz D. Bergmann S. Didiasova M. Giaimo B. D. Borggrefe T. Mieth M. (2016). Host-derived extracellular RNA promotes adhesion of *Streptococcus pneumoniae* to endothelial and epithelial cells. *Sci. Rep.* 6:37758. 10.1038/srep37758 27892961 PMC5125276

[B71] Zysk G. Schneider-Wald B. K. Hwang J. H. Bejo L. Kim K. S. Mitchell T. J. (2001). Pneumolysin is the main inducer of cytotoxicity to brain microvascular endothelial cells caused by *Streptococcus pneumoniae*. *Infect. Immun.* 69 845–852. 10.1128/IAI.69.2.845-852.2001 11159977 PMC97961

